# Malaria and anaemia among children in two communities of Kumasi, Ghana: a cross-sectional survey

**DOI:** 10.1186/1475-2875-5-105

**Published:** 2006-11-09

**Authors:** Lisa A Ronald, Sarah L Kenny, Eveline Klinkenberg, Alex O Akoto, Isaac Boakye, Guy Barnish, Martin J Donnelly

**Affiliations:** 1 Liverpool School of Tropical Medicine, Pembroke Place, Liverpool, UK; 2 International Water Management Institute (IWMI), Accra, Ghana; 3 Kwame Nkrumah University of Science and Technology and Komfo Anokye Teaching Hospital, Kumasi, Ghana

## Abstract

**Background:**

A survey in Kumasi, Ghana found a marked *Plasmodium falciparum *prevalence difference between two neighbouring communities (Moshie Zongo and Manhyia). The primary objective of this follow-up study was to determine whether this parasite rate difference was consistent over time. Secondary objectives were to compare prevalences of clinical malaria, anaemia, intestinal parasite infections, and malnutrition between these communities; and to identify potential risk factors for *P. falciparum *infection and anaemia.

**Methods:**

A cross-sectional house-to-house survey of *P. falciparum *parasitaemia, clinical malaria, anaemia, anthropometric indices, and intestinal helminths was conducted in April-May 2005. Data collection included child and household demographics, mosquito avoidance practices, distance to nearest health facility, child's travel history, symptoms, and anti-malarial use. Risk factors for *P. falciparum *and anaemia (Hb < 11 g/dl) were identified using generalized linear mixed models.

**Results:**

In total, 296 children were tested from 184 households. Prevalences of *P. falciparum*, clinical malaria, anaemia, and stunting were significantly higher in Moshie Zongo (37.8%, 16.9%, 66.2% and 21.1%, respectively) compared to Manhyia (12.8%, 3.4%, 34.5% and 7.4%). Of 197 children tested for helminths, four were positive for *Dicrocoelium dendriticum*. Population attributable risks (PAR%) of anaemia were 16.5% (*P. falciparum*) and 7.6% (malnutrition). Risk factors for *P. falciparum *infection were older age, rural travel, and lower socioeconomic status. Risk factors for anaemia were *P. falciparum *infection, Moshie Zongo residence, male sex, and younger age.

**Conclusion:**

Heterogeneities in malariometric indices between neighbouring Kumasi communities are consistent over time. The low helminth prevalence, and the twofold higher PAR% of anaemia attributable to *P. falciparum *infection compared to malnutrition, indicate the importance of malaria as a cause of anaemia in this urban population.

## Background

Although often considered a predominantly rural disease in Africa, malaria represents a leading cause of morbidity and mortality among many urban African populations [1]. With an estimated 200 million urban residents currently at risk for malaria [2] and a projected doubling of the African urban population by 2030 [3], an urgent need to identify risk factors for malaria in urban settings has been identified [1,4].

In Africa, malaria transmission is generally lower in urban compared with rural areas [5,6]. This is largely attributed to such factors as lower vector density, higher human density, better quality housing and improved drainage in urban areas [7]. Urban populations are also better able to access healthcare facilities and consequently suffer lower malaria morbidity and mortality [5]. Malaria risk is often unevenly distributed across urban environments, with heterogeneities in malariometric indices measured between and within many cities [8,9]. Evidence is also accumulating that in urban areas, the poor may be most vulnerable to the impacts of malaria [8]. While urban malaria should be amenable to targeted prevention and control programmes, a better understanding of the individual, environmental, and community-based determinants within specific communities is needed [1,2].

A baseline survey of malaria in children aged 6 months to 5 years, conducted in 2002–3 in Kumasi, Ghana, found marked prevalence differences between communities [8]. Proximity to urban agriculture was not significantly associated with malaria prevalence, suggesting the importance of other risk factors. This study reports findings from a follow-up cross-sectional survey comparing two neighbouring communities (Moshie Zongo and Manhyia), which in the previous survey were found to have *Plasmodium falciparum *parasite rates of 32.7% and 3.8%, respectively. The primary objective of this study was to determine whether the previously observed marked difference in parasite rates between the two communities was maintained. The secondary objectives were to compare prevalences of clinical malaria, anaemia, intestinal helminths, and malnutrition between these communities, and to identify risk factors for *P. falciparum *infection and anaemia.

## Methods

Kumasi is the second-largest city in Ghana, with a population of 1.2 million [10]. Located in the Ashanti region of central Ghana, the climate is semi-humid tropical, with peak rainfall between April and June.

### Sample selection

A cross-sectional house-to-house survey was carried out in Moshie Zongo and Manhyia during April and May 2005. Sampling was conducted between 14:00 and 19:00 and alternated on an approximately weekly basis between communities. Cluster starting points were identified based on proximity to specific landmarks (i.e., roads, intersections, and prominent buildings), with the whole area of each community included to gain a geographically representative sample of the community. A house was defined as any housing structure where one or more families were residing. The first house was selected randomly in each cluster, followed by the selection of every fifth house until a minimum cluster target of 10 children was reached. Voluntary written consent was obtained from primary carers prior to data collection. All children in the target age range present at the time of sampling were eligible to participate. The location of each sampled house was recorded using a handheld GPS receiver. A minimum sample size of 108 children per community was calculated using EpiInfo v.3.2.2 (CDC, Atlanta), based on detection of a 25% difference in *P. falciparum *parasite rates between the two communities (with a 95% confidence interval and power of 90%) and multiplied by 2 for a design effect. The study received ethical approval from the Liverpool School of Tropical Medicine and the Faculty of Medicine, Kwame Nkrumah University of Science and Technology, Kumasi.

### Data collection

Data about household demographics, assets and house construction, together with the carer's knowledge about malaria and use of mosquito avoidance methods, were collected by questionnaire. Age, sex, recent rural travel, symptom history, and recent anti-malarial use were recorded for each child. The presence of *P. falciparum *parasitaemia was determined using a Rapid Diagnostic Test (RDT) (Paracheck^®^, Orchid Biomedical Systems, India). Haemoglobin (Hb) concentrations were measured using a HemoCue system (HemoCue, Sweden). Axillary temperature was measured by digital thermometer. Clinical malaria was defined as presence of *P. falciparum*, with a current axillary temperature of ≥ 37.5°C and/or a reported fever history during the previous 48 hours. Anthropometric measurements included height, weight, and mid-upper arm circumference (MUAC). Stool samples were collected at the time of the haematological and anthropometric sampling or the following day. Faecal samples were preserved in 10% formal saline. A volumetric dilution procedure was used to analyse the stool samples for intestinal helminths [11].

Socio-economic status (SES) was calculated for each household using World Bank asset scores for Ghana, based on ownership of a television, radio, refrigerator, car, motorcycle, bicycle, farm, electricity in house, ceiling, floor cover, and number of individuals/sleeping room [12]. The anthropometric indices height-for-age (HA), weight-for-age (WA), weight-for-height (WH), and MUAC-for-height were expressed as Z-scores in relation to the 1978 CDC/WHO normalized version of the 1977 NCHS reference curve (EpiInfo v3.3, CDC). Children were classified as stunted, underweight, wasted, or having low MUAC-for-height if the HA, WA, WH, or MUAC-for height scores were < -2 below the reference mean, respectively. A child was identified as being malnourished if s/he scored < -2 in one of the HA, WA, or WH indices.

A list of all government hospitals and primary care clinics in the surrounding area was obtained from the Kumasi Metro Director of Health and verified by the Manhyia District Hospital Administrator (the main catchment hospital for both communities). The location of each health facility was mapped using a GPS. Straight-line distance to the nearest health facility was used as a proxy for access to formal healthcare services and was calculated using ArcInfo v7.2.1 (ESRI Inc., USA).

### Data analysis

Statistics were calculated using SPSS v11.5 (USA) and SAS v8.2 (SAS Institute Inc, Cary, USA). The proportion of anaemia cases attributed to *P. falciparum *infection and malnutrition were estimated as population attributable risk percentages (PAR%) with 95% confidence intervals (CI) [13]. Univariate significance tests used were Pearson's chi-square or Fisher's Exact test (for categorical variables), and for continuous variables, independent samples t-test or Mann-Whitney U test. Generalized linear mixed models were run with PROC MIXED, incorporating a SAS macro (Glimmix 800) allowing a logistic link function. Outcomes tested were presence of *P. falciparum *infection and Hb < 11 g/dl. Variables not highly predictive of outcome variables (p > 0.1) in unadjusted models were excluded from further modelling. For all models, household was included as a random effect. Final adjusted models included all variables significant at p < 0.05.

## Results

Table 1 summarizes characteristics of the sampled households (97 in Manhyia, 87 in Moshie Zongo). Household characteristics were markedly different between the communities, with the exception of reported use of bednets, housescreens, and mosquito repellents/coils/sprays (p > 0.05).

**Table 1:** Characteristics of sampled households, by community (N = 184)

	Manhyia (n = 97)	Moshie Zongo (n = 87)	p-value^1^
Caregiver education ≥ middle school, n (%)	75 (77.3%)	28 (32.2%)	< 0.001***
Ethnicity Northerner/Immigrant, n (%)	18 (18.6%)	65 (74.7%)	< 0.001***
Socioeconomic score, mean (standard deviation)^2^	2.07 (0.73)	1.25 (0.70)	< 0.001***
House without ceiling, n (%)	5 (5.2%)	17 (19.5%)	0.003**
Number of people per sleeping room, median (range)	4 (2,9)	5 (1,11)	0.020*
Reported use of mosquito repellents/coils/spray, n (%)	63 (64.9%)	67 (77.0%)	0.073
Reported use of bednet, n (%)	15 (15.5%)	21 (24.1%)	0.139
Reported use of housescreens, n (%)	11 (11.3%)	4 (4.6%)	0.095
Metres to nearest health facility, median (range)	131 (26,345)	237 (74,436)	< 0.001***
Metres to nearest government hospital, median (range)	730 (241,990)	2344 (1958,2550)	< 0.001***

1. * p < 0.05, ** p < 0.01, *** p < 0.001

2. Calculated with World Bank asset scores for Ghana [12]

A total of 296 children were tested, 148 in each community (Table 2). Sample composition (sex ratio and median age) did not significantly differ between the communities (p > 0.05). Prevalences of *P. falciparum *(*X*^2 ^= 24.45, p < 0.001), clinical malaria (*X*^2 ^= 14.84, p < 0.001), and anaemia (*X*^2 ^= 29.85, p < 0.001) were higher, and mean Hb levels lower (p < 0.001), in Moshie Zongo compared to Manhyia. There were few cases of moderate to severe anaemia (< 3% prevalence in each community). More children from Moshie Zongo were stunted (*X*^2 ^= 11.26, p = 0.001), had a reported history of diarrhoea (*X*^2 ^= 8.64, p = 0.003), and had reportedly taken malaria medication in the previous two weeks (*X*^2 ^= 3.95, p = 0.047). Fewer than 5% in either community had travelled recently to a rural area. Stools were examined for the first 197 children surveyed. With the exception of 4 children from neighbouring households in Manhyia who had *Dicrocoelium dendriticum *infections, no helminth eggs were found. As a consequence, stool samples were not collected from the remaining 99 children.

**Table 2:** Test results of children sampled, by community (N = 296)

	Manhyia (n = 148)	Moshie Zongo (n = 148)	p-value^1^
Female sex, n (%)	74 (50.0%)	84 (56.8%)	0.244
Age in years, median (range)	5 (1, 9)	5 (1, 9)	0.571
Presence of *P. falciparum *parasitaemia, n (%)	19 (12.8%)	56 (37.8%)	< 0.001***
Axillary temp ≥ 37.5°C or reported fever past 48 hours	28 (18.9%)	50 (33.8%)	0.004**
Clinical malaria, n (%) ^2^	5 (3.4%)	25 (16.9%)	< 0.001***
Haemoglobin level in g/dl, mean (standard deviation)	11.3 (1.28)	10.5 (1.25)	< 0.001***
Any anaemia: Hb < 11 g/dl, n (%)	51 (34.5%)	98 (66.2%)	< 0.001***
Moderate to severe anaemia: Hb < 8 g/dl, n (%)	3 (2.0%)	4 (2.7%)	1.000
Malnourished (stunted, wasted, or underweight), n (%)	28/147 (19.0%)	40/147 (27.2%)	0.097
Stunted (height-for-age Z-score < -2 ref mean), n (%)	11 (7.4%)	31/147 (21.1%)	0.001**
Underweight (weight-for-height Z-score < -2), n (%)	15 (10.1%)	19/147 (12.9%)	0.453
Wasted (weight-for-age Z-score < -2), n (%)	8/147 (5.4%)	8/146 (5.5%)	0.989
Low MUAC-for-height (Z-score < -2), n (%)^3^	7 (4.7%)	9/147 (6.1%)	0.597
Intestinal helminths, n (%)^4^	4/98 (4.1%)	0/99 (0.0%)	0.059
Reported history of diarrhoea in past 48 hours, n (%)	28 (18.9%)	50/147 (34.0%)	0.003**
Rural travel in past 3 weeks, n (%)	4 (2.7%)	6 (4.1%)	0.520
Reported malaria medication in past 2 weeks, n (%)	31 (20.9%)	46 (31.1%)	0.047*

1. * p < 0.05, ** p < 0.01, *** p < 0.001

2. *P. falciparum *infection with an axillary temp ≥ 37.5°C or reported fever past 48 hours

3. MUAC = mid upper arm circumference

4. Four cases of *Dicrocoelium dendriticum*

Significantly higher prevalence of *P. falciparum *was associated with low carer education, Northerner/Immigrant ethnicity, low SES, using coils/sprays/repellents, greater distance from a health facility, older age, and recent rural travel (Table 3). The same variables were significantly associated with higher anaemia prevalence, with the exception of younger age, and the addition of male sex, stunting, *P. falciparum*, and clinical malaria. No significant differences in *P. falciparum *or anaemia prevalence were observed with reported use of bednets or housescreens, recent antimalarial medication, wasting, underweight, or low MUAC-for-height. The proportion of fever cases with *P. falciparum *infection were 17.9% in Manhyia (5/28 fever cases) and 50.0% in Moshie Zongo (25/50). The PAR% of anaemia attributable to *P. falciparum *infection was 16.5% (95%CI: 9.5–23.3), and to malnutrition was 7.6% (95%CI: 1.4–13.9).

**Table 3:** Factors associated with *P. falciparum *infection and anaemia (Hb < 11 g/dl), significant associations in univariate tests (N = 296)^1^

**Variables tested **	**Prevalence of *P. falciparum *infection (95% CI)**		**Prevalence of Hb < 11 (95% CI)**	
Education ≥ middle school				
No (n = 132)	40.9%	(32.5, 49.3)***	63.6%	(55.4, 71.8)***
Yes (n = 164)	12.8%	(7.7, 17.9)	39.6%	(32.1, 47.1)
Ethnic group				
Akan/Ga/Ewe (n = 167)	16.2%	(10.6, 21.8)***	35.9%	(28.6, 43.2)***
North/Immigrant (n = 129)	37.2%	(28.9, 45.5)	69.0%	(61.0, 77.0)
Socioeconomic score^2^				
Lowest (n = 80)	47.5%	(36.6, 58.4)***	67.5%	(57.2, 77.8)**
Middle (n = 109)	26.6%	(18.3, 34.9)	47.7%	(38.3, 57.1)
Highest (n = 107)	7.5%	(2.5, 12.5)	40.2%	(30.9, 49.5)
Use repellents/coils/sprays				
No (n = 83)	15.7%	(7.9, 23.5)*	41.0%	(30.4, 51.6)*
Yes (n = 213)	29.1%	(23.0, 35.2)	54.0%	(47.3, 60.7)
Nearest health facility^3^				
< 150 m (n = 97)	12.4%	(5.8, 19.0)***	45.4%	(35.5, 55.3)**
150–250 m (n = 112)	26.8%	(18.6, 35.0)	42.9%	(33.7, 52.1)
≥ 250 m (n = 87)	37.9%	(27.7, 48.1)	65.5%	(55.5, 75.5)
Child sex				
Male (n = 138)	24.6%	(17.4, 31.8)	58.0%	(49.8, 66.2)*
Female (n = 158)	25.9%	(19.1, 32.7)	43.7%	(36.0, 51.4)
Child age group				
1–3 years (n = 108)	15.7%	(8.8, 22.6)**	61.1%	(51.9, 70.3)**
4–6 years (n = 106)	34.0%	(25.0, 43.0)	51.9%	(42.4, 61.4)
7–9 years (n = 82)	26.8%	(17.2, 36.4)	34.1%	(23.8, 44.4)
Rural travel, past 3 weeks				
No (n = 286)	23.8%	(18.9, 28.7)**	49.3%	(43.5, 55.1)
Yes (n = 10)	70.0%	(41.6, 98.4)	80.0%	(55.2, 100)
Malnourished(stunted, wasted or underweight)				
No (n = 226/294)	24.8%	(19.2, 30.4)	46.5%	(40.0, 53.0)*
Yes (n = 68/294)	26.5%	(16.0, 37.0)	63.2%	(51.7, 74.7)
Stunted (height-for-age Z-score < -2)				
No (n = 253/295)	23.7%	(18.5, 28.9)	47.0%	(40.8, 53.2)**
Yes (n = 42/295)	33.3%	(19.0, 47.6)	71.4%	(57.7, 85.1)
*P. falciparum *infection				
No (n = 221)	n/a		42.1%	(35.6, 48.6)***
Yes (n = 75)			74.7%	(64.9, 84.5)
Clinical malaria^4^				
No (n = 266)	n/a		47.0%	(41.0, 53.0)**
Yes (n = 30)			80.0%	(65.7, 94.3)

1. *p < 0.05, **p < 0.01, ***p < 0.001

2. Based on World Bank asset scores for Ghana [12]: lowest (scores = 0,1), middle (2), highest (3.4)

3. Health facility = government hospital or primary care clinic

4. Clinical malaria: *P. falciparum *with reported fever in past 48 hours and/or current axillary temperature ≥ 37.5°C

In adjusted models (Table 4), risk factors for *P. falciparum *infection included being older (OR = 1.28 per 1-year increase), recent rural travel (OR = 23.45), and lower SES (OR = 3.95 per 1-unit decrease). Significant risk factors for anaemia were *P. falciparum *infection (OR = 3.92), Moshie Zongo residence (OR = 3.76), male sex (OR = 2.16), and younger age (OR = 0.79 per 1-year increase).

**Table 4:** Unadjusted and adjusted odds ratios (with 95% confidence intervals)^1 ^for predictors of *P. falciparum *infection and Hb < 11 g/dl

	Unadjusted models^2 ^OR (95% CI)	Adjusted- final model OR (95% CI)
**Model 1: *P. falciparum *infection**		

Rural travel past 3 weeks	12.80 (2.20, 74.46)**	23.45 (3.28, 167.75)**
Socioeconomic score^2^	3.22 (1.95, 5.32)***	3.95 (2.26, 6.90)***
Child age, in years	1.22 (1.08, 1.38)**	1.28 (1.13, 1.45)***

**Model 2: Hb < 11 g/dl**		

*P. falciparum *infection	3.94 (2.17, 7.15)***	3.92 (2.04, 7.54)***
Moshie Zongo residence	3.80 (2.29, 6.30)***	3.76 (2.16, 6.56)***
Child male	1.83 (1.13, 2.97)*	2.16 (1.28, 3.66)**
Child age, in years^2^	0.84 (0.76, 0.93)***	0.79 (0.71, 0.88)***

^1. ^Households were included as a random effect for all models, *p < 0.05, **p < 0.01, ***p < 0.001

^2. ^Child age (from 1 to 9 years) and socioeconomic score (from 3: highest to 0: lowest) are included in models as continuous variable

## Discussion

This study demonstrated that the higher prevalence of *P. falciparum *among children in Moshie Zongo compared to neighbouring communities is temporally stable and not an artefact of a single study period [8]. The *P. falciparum *rate in Moshie Zongo was higher than recently reported rates in other urban areas; Kampala [14], Coutonou [15] and Dar es Salaam [16], though lower than rates reported in Ouagadougou [17]. Parasite rates from these latter surveys were obtained through primary school parasitaemia surveys and represent aggregated rates for communities in each school's catchment area. Further breakdown of rates by residence in Coutonou indicated a large parasitaemia rate difference (27.3% and 9.2%) between two intermediate zone areas [15]. Heterogeneities between neighbouring communities have also been reported in Brazzaville, where rates ranged from 5.6% to 78.9% among children aged 5–9 years in 4 urban communities [9]. This study adds to the growing body of evidence that malaria transmission can vary widely across cities and highlights the importance of targeting interventions towards specific communities [1].

The finding that recent rural travel was associated with increased *P. falciparum *prevalence has been similarly reported among other urban populations, including Dar es Salaam [16], Abidjan [18], Lusaka [19], and previously in Kumasi [8]. However, few children from either study community had travelled recently, which suggests that most infections were obtained through local transmission. High *P. falciparum *prevalence has been recorded in rural areas of the surrounding Ashanti region [20] and Northern Ghana [21]. Urban residents who travel to these areas could be at high risk for infection, as well as at higher risk for more severe outcomes following infection if functional immunity is inadequately developed, and may become sources of infection when back home [22]. Promoting increased vigilance in the use of mosquito avoidance methods and/or prophylactic treatment among urban residents who travel to endemic rural areas could be an effective target for reducing some subsequent transmission within urban areas.

Two-thirds of the Moshie Zongo children sampled in our study were anaemic (an approximately two-fold higher prevalence than found among children in Manhyia). Prevalence of severe anaemia was low in both communities. When restricted to children under 5 years, the anaemia prevalence in the present study was comparable to the previous Kumasi survey [8], and to the 2003 Ghana Demographic and Health Survey for urban residents (67.8%) [10]. Demonstration of heterogeneity in anaemia rates within cities underscores the importance of targeting anaemia prevention programs to both rural and urban populations, and of targeting interventions to specific urban communities.

Anaemia is often caused by a combination of malnutrition, helminth infections, infectious diseases, and haemoglobinopathies [23,24]. Significant reductions in anaemia prevalence have been demonstrated following malaria control programs in stable endemic areas, and usefulness of anaemia as an indicator of malaria burden and malaria control progress will depend on its specificity [24]. In lower transmission urban areas, malaria may make a less significant contribution to anaemia than other factors. The PAR% estimates indicated that approximately one-sixth of anaemia cases were attributable to *P. falciparum *infections, and *P. falciparum *infection was strongly associated with anaemia after adjustment for other factors. This suggests that *P. falciparum *infection is an important determinant of anaemia among these Kumasi children and that anaemia may represent a useful indicator for malaria control progress in these communities.

In comparison with these Ghanaian study populations, a higher prevalence of stunting but similar prevalence of wasting was found among urban Zairian children [25]. In these children from Kumasi, malnutrition (stunting, in particular) was significantly associated with anaemia in univariate tests. Stunting has been previously associated with more severe occurrence of malarial anaemia [26], and it is hypothesized that nutritional inadequacies causing stunting and underweight also impair host immunity, further exacerbating the effects of malaria [26,27]. In contrast, the low prevalence of intestinal helminths was surprising, compared to higher rates reported in other urban areas [25]. Parasitology laboratory records (1999–2005) from the Kumasi Komfo Anokye Teaching Hospital revealed similar low rates of intestinal helminths: of the 1,181 children aged 1–9 years tested for intestinal parasites over this period, only 164 (13.9%) were positive for one or more parasites (including 10 with hookworm, 8 *A. lumbricoides*, and 1 *T. trichiura*). Kumasi is exposed to problems of crowding and sewage disposal, which are often associated with increased risk of helminth infections [28]. One explanation for the low prevalence of helminth infections in Kumasi could be that many parents may be regularly de-worming their children (as reported anecdotally), however, further investigation is needed to confirm these results.

The Paracheck-Pf RDTs used in this survey detect the presence ofHistidine Rich Protein 2 (HRP-2), a water soluble protein produced by asexual stages and young gametocytes of *P. falciparum *[29]. The benefits of using Paracheck Pf RDTs in community-based surveys are their high sensitivity at low levels of parasitaemia (> 150 parasites/μl) and high specificity to *P. falciparum*, and that they provide results quickly (within 15 minutes) [30]. One limitation of HRP-2-based RDTs is that HRP-2 has been shown to persist and be detectable after the clinical symptoms of malaria have disappeared and the parasites have been cleared [31]. Surveys using RDTs detecting HRP-2 may, therefore, overestimate prevalence of current infection, representing instead a combined prevalence of current and recent infections.

Twenty-five percent (25.3%) of children with *P. falciparum *infection and 36.7% of children with clinical malaria had reported taking an antimalarial within the previous 2 weeks. The proportion of *P. falciparum *cases with reported recent antimalarial use was higher in Moshie Zongo (28.6%) than Manhyia (15.8%). It is possible that some false positives were detected due to residual HRP-2, while recall bias may also have resulted in overestimation of reported antimalarial use. However, this evidence also suggests that treatments being used by some children may not be effective in clearing parasitaemia. Home treatment or reliance on informal drug shops as a primary treatment method for child malaria is reported among many urban populations [32,33]. In eastern Uganda, treatments and dosages recommended by drug shops and government health units were often incorrect, and poor caregiver compliance with prescribed dosages was common [34]. Poor quality drugs with insufficient active ingredients have also been found among informal drug vendors in Cameroon [35]. In addition, only 50% of fever cases in Moshie Zongo and 18% in Manhyia had a *P. falciparum *infection. As many urban caregivers in Ghana report treating all child fevers with antimalarials [10], this suggests some over-utilization of antimalarials by children without *P. falciparum *infection in our study communities. Further investigation is needed to determine the quality of drugs being sold in these areas and to assess patterns of drug utilization by caregivers.

In addition, this study highlights the vulnerability of the urban poor to the impact of malaria, as a strong association was found between SES and disease prevalence. Disaggregating the mechanisms through which poverty acts is challenging due to the complex interactions of factors [36]. The SES measure was a composite and could partly reflect greater exposure to infected mosquitoes through poor quality house construction, or greater infection risk due to crowding [36]. Among sampled households, Moshie Zongo residents lived further on average to the nearest health facility, and more than 1 km further to the nearest government hospital. Studies in rural areas have found an association with distance to health clinics and treatment patterns for child malaria [37,38], however, the influence of distance on treatment choice in urban areas is not well-documented. It is possible that distance to health facilities in our study reflected a proxy measure for other factors which differed between the two communities. Furthermore, sample sizes were calculated to detect a difference in *P. falciparum *prevalence, and as a consequence, this study may have more limited power to assess differences in other factors between the communities. No data were collected to differentiate clinics by ownership type, treatment costs, or the range of services provided by each clinic; however, all of the clinics operating in Moshie Zongo were private clinics, which could have different treatment fees than government facilities. Good access to health services and timely provision of effective drug treatment is a self-targeting intervention, yet the costs of treatment and prevention often preclude the poorest people from using them [39]. Greater distance to affordable health services could result in delayed seeking of treatment. Further research is needed to determine if treatment-seeking behaviours in these communities differ and to what extent these behaviours are associated with availability of different types of health services.

## Conclusion

Heterogeneities in malariometric indices between neighbouring urban communities of Kumasi are consistent over time. The low prevalence of intestinal helminths, and the twofold higher PAR% of anaemia attributable to *P. falciparum *infection compared to malnutrition, indicate the importance of malaria as a cause of anaemia in this urban population. Further investigation is needed to determine if treatment-seeking behaviours in these two communities differ and to what extent behaviours are associated with availability of different types of health services.

## Authors' contributions

LAR helped to design the study, performed data collection, analysis and drafted the manuscript. SLK helped to design the study, performed data collection and analysis. EK helped to design the study and assisted with the analysis. AOA helped to design the study and supervised data collection. IB performed data collection. GB designed and supervised the study. MJD conceived of the study, and participated in its design and coordination. All authors read and approved the final manuscript.

## Competing interests

The author(s) declare that they have no competing interests.
